# Selective Isolation of TOP3B•mRNA Covalent Intermediates Using Denaturing Oligo-dT Pulldown

**DOI:** 10.21769/BioProtoc.5627

**Published:** 2026-03-05

**Authors:** Julia E. Warrick, Michael G. Kearse

**Affiliations:** Department of Biological Chemistry and Pharmacology, Center for RNA Biology, The Ohio State University, Columbus, OH, USA

**Keywords:** mRNA pulldown, Post-transcriptional gene regulation, RNA-based mechanisms of disease, RNA-binding protein, Slot blot, Translational control

## Abstract

The deletion and mutation of Topoisomerase 3β (TOP3B) is linked to multiple neurological disorders and is the only known topoisomerase that is also catalytically active on RNA in vitro and in cells. Uniquely, TOP3B is primarily localized to the cytoplasm, binds to open reading frames of mRNA, and regulates mRNA stability and translation in a transcript-specific manner. A common approach for studying TOP3B activity in cells is immunodetection of TOP3B•RNA covalent intermediates after bulk RNA isolation. However, in this approach, the RNA species is unknown and is not selective for the major TOP3B substrate, mRNA. In this protocol, we describe a recently developed and optimized protocol for capturing TOP3B•mRNA covalent intermediates using oligo-dT isolation of mRNA under protein-denaturing conditions. Covalent intermediates are then detected by a dual membrane slot blotting strategy with nitrocellulose and positively charged nylon membranes. Nitrocellulose membrane-bound TOP3B•mRNA covalent intermediates are analyzed by immunodetection, and nylon membrane-bound free mRNA is stained with methylene blue. The protocol detailed below has been validated with wildtype and mutant 3xFLAG-tagged TOP3B expressed in Neuro2A cells, with additional optimization for slot blotting using recombinant EGFP.

Key features

• This protocol is optimized for isolation of TOP3B•mRNA covalent intermediates from cultured mammalian cells.

• Slot blotting allows for higher throughput and sensitive detection of TOP3B•mRNA covalent intermediates and allows for free mRNA to serve as a loading control.

• Alternative to laborious extraction methods that do not select for TOP3B covalently linked to mRNA over other RNA species.

• Can be completed in two days (not including variable time for mammalian cell sample collection).

## Graphical overview


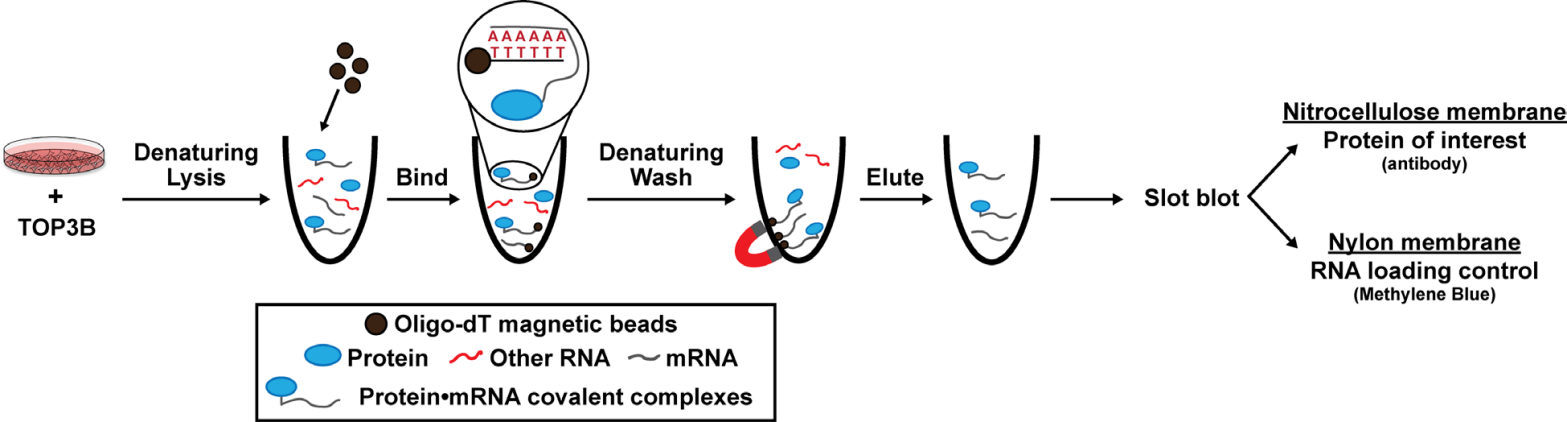


**Schematic of denaturing oligo-dT isolation of TOP3B•mRNA covalent intermediates**

## Background

RNA-binding proteins (RBPs) have diverse functions that are critical for every level of gene expression and are linked to multiple human diseases [1,2]. Deletion and mutation of Topoisomerase 3β (TOP3B) are linked to schizophrenia, autism spectrum disorder, and intellectual disability [3–7]. Notably, TOP3B is the only human topoisomerase that functions on both DNA and RNA, and multiple reports suggest that mRNA is its primary substrate [4,8,9]. Unlike most other RBPs, but similar to other topoisomerases, TOP3B forms an intermediate that is covalently linked to the 5′ phosphate of its substrate RNAs during the catalytic cycle. CLIP-based studies have shown that TOP3B primarily binds to the open reading frame (ORF) of mRNAs [4,9], which is common for RBPs that have a role in mRNA translation and post-transcriptional gene regulation. Additionally, recent ribosome profiling data indicated that TOP3B regulates the translation of some mRNAs by acting as a traditional RBP where catalytic activity is not necessary, while other mRNAs are regulated by TOP3B in a catalytic activity-dependent manner [9]. Therefore, it is important to distinguish the mRNA-binding capability from catalytic cleavage activity of TOP3B to identify which steps in the cycle may be affected by de novo mutations present in the human population and to understand mechanisms of translational control governed by TOP3B. However, published cell-based assays are limited due to laborious cesium chloride gradients and multiple large-scale extractions from the interface of acidic guanidinium thiocyanate-phenol-chloroform-based approaches (e.g., TRIzol, TRI Reagent) and depend on assaying unidentified RNAs from cells [10–13]. As most data in the field suggest that mRNA is the primary target of TOP3B, there is a need to develop an approach to assess TOP3B activity on mRNA over other RNA species.

Here, we provide a published method to selectively evaluate covalently linked TOP3B•mRNA complexes using denaturing oligo-dT pulldown and slot blotting [14]. We ectopically express wildtype (WT) and mutant 3xFLAG-tagged TOP3B in cells via plasmid transfection and incubate the cell lysate with oligo-dT magnetic beads to selectively bind the poly(A) tail of mRNA. Lysis and wash conditions are denaturing, allowing the removal of any RBPs that are not covalently linked to mRNA and the retention of TOP3B•mRNA covalent intermediates. We then evaluate the TOP3B•mRNA intermediate levels by slot blotting for the 3xFLAG tag. As validation, we show two mutants that produce decreased and increased intermediate levels compared to the wildtype TOP3B [10,14]. Intermediate levels from a catalytically inactive TOP3B (Y336F), which can bind mRNA but cannot form covalent intermediates, are markedly reduced. Conversely, intermediate levels from a “self-trapping” mutant TOP3B (R338W), which causes accumulation of unresolved TOP3B•mRNA intermediates by preventing the rejoining step after substrate cleavage, are robustly detected. We have recently published and further validated this method using TOP3B tagged on either the N- or C-terminus, as well as with domain deletions and point mutations [14].

## Materials and reagents

**Biological materials**

1. Neuro-2A cells (ATCC, catalog number: CCL-131)

**Reagents**

1. 1 M Tris-HCl, pH 7.4 (Apex Bioresearch Products, catalog number: 18-190)

2. 2 M lithium chloride (LiCl) (Sigma, catalog number: L7026-1L)

3. Lithium dodecyl sulfate (LiDS) (Sigma, catalog number: L9781-50G)

4. 500 mM EDTA, pH 8.0 (Millipore, catalog number: 324504-500mL)

5. 1 M DTT (Thermo Scientific, catalog number: R0861)

6. Oligo-dT magnetic beads (NEB, catalog number: S1419S)

7. 1% (w/v) methylene blue (Thermo Scientific, catalog number: 042771.AP)

8. 17.4 M glacial acetic acid (Fisher Chemical, catalog number: A38C-212)

9. 3 M sodium acetate, pH 5.5 (Invitrogen, catalog number: AM9740)

10. 1× phosphate-buffered saline (PBS), pH 7.4 (Quality Biological, catalog number: 114-058-131)

11. 10× Tris-buffered saline (TBS), pH 7.4 (Quality Biological, catalog number: 351-086-151)

12. 100% Tween-20 (VWR, catalog number: 0777-4L)

13. Instant non-fat dry milk (Quality Biological, catalog: A614-1001) or equivalent

14. Sodium azide (Sigma, catalog number: S2002-5G)

15. Mouse anti-FLAG M2 primary antibody (Sigma, catalog number: F3165-1mg)

16. Rabbit anti-GFP (D5.1) primary antibody (Cell Signaling Technologies, catalog number: 2956)

17. HRP-conjugated goat anti-mouse IgG (H+L) (Invitrogen, catalog number: 31430)

18. HRP-conjugated goat anti-rabbit IgG (H+L) (Invitrogen, catalog number: 31460)

19. SuperSignal West Pico PLUS chemiluminescent substrate (Invitrogen, catalog number: 34578)

20. Recombinant EGFP (Abcam, catalog number: ab134853)

21. Plasmid pCMV6/TOP3B (WT)-3xFLAG (Addgene, plasmid number: 249681)

22. Plasmid pCMV6/TOP3B (Y336F)-3xFLAG (Addgene, plasmid number: 249682)

23. Plasmid pCMV6/TOP3B (R338W)-3xFLAG (Addgene, plasmid number: 249683)

23. Ultrapure water (from Millipore ultrapure water system or equivalent)

**Solutions**

1. 1 M DTT (see Recipes)

2. 10% (w/v) LiDS (see Recipes)

3. Oligo-dT lysis buffer (see Recipes)

4. Oligo-dT wash I buffer (see Recipes)

5. Oligo-dT wash II buffer (see Recipes)

6. Oligo-dT low salt buffer (see Recipes)

7. Oligo-dT elution buffer (TE) (see Recipes)

8. Methylene blue staining solution (see Recipes)

9. TBST (see Recipes)

10. 5% (w/v) non-fat dry milk (see Recipes)

11. 20 mM Tris-HCl, pH 7.4 (see Recipes)

12. 2% (w/v) sodium azide (see Recipes)

13. Primary antibody solution (see Recipes)

14. Secondary antibody solution (see Recipes)

**Recipes**

**1. 1 M DTT**

**Table:** 

Reagent	Final concentration	Quantity or volume
DTT	1M	1.55 g
Ultrapure water	n/a	To 10 mL
Total	n/a	10 mL

**2. 10% (w/v) LiDS**

**Table:** 

Reagent	Final concentration	Quantity or volume
LiDS	10% (w/v)	50 g
Ultrapure water	n/a	To 500 mL
Total	n/a	500 mL

*Safety note: Wear a mask to avoid inhaling the reagent.*

**3. Oligo-dT lysis buffer**

**Table:** 

Reagent	Final concentration	Quantity or volume
1 M Tris-HCl, pH 7.4	100 mM	30 mL
8 M LiCl	500 mM	18.75 mL
10% LiDS (w/v)	0.5% (w/v)	15 mL
500 mM EDTA, pH 8.0	1 mM	600 μL
Ultrapure water	n/a	235.65 mL
Total	n/a	300 mL

**4. Oligo-dT wash I buffer**

**Table:** 

Reagent	Final concentration	Quantity or volume
1 M Tris-HCl, pH 7.4	20 mM	6 mL
8 M LiCl	500 mM	18.75 mL
10% (w/v) LiDS	0.1% (w/v)	3 mL
500 mM EDTA, pH 8.0	1 mM	600 μL
Ultrapure water	n/a	271.65 mL
Total	n/a	300 mL

**5. Oligo-dT wash II buffer**

**Table:** 

Reagent	Final concentration	Quantity or volume
1 M Tris-HCl, pH 7.4	20 mM	6 mL
8 M LiCl	500 mM	18.75 mL
500 mM EDTA, pH 8.0	1 mM	600 μL
Ultrapure water	n/a	274.65 mL
Total	n/a	300 mL

**6. Oligo-dT low salt buffer**

**Table:** 

Reagent	Final concentration	Quantity or volume
1 M Tris-HCl, pH 7.4	20 mM	3 mL
8 M LiCl	200 mM	3.75 mL
500 mM EDTA, pH 8.0	1 mM	300 μL
Ultrapure water	n/a	142.95 mL
Total	n/a	150 mL

**7. Oligo-dT elution buffer (TE)**

**Table:** 

Reagent	Final concentration	Quantity or volume
1 M Tris-HCl, pH 7.4	20 mM	6 mL
500 mM EDTA, pH 8.0	1 mM	600 μL
Ultrapure water	n/a	293.4 mL
Total	n/a	300 mL

**8. Methylene blue staining solution**

**Table:** 

Reagent	Final concentration	Quantity or volume
1% (w/v) methylene blue	0.2% (w/v)	10 mL
17.4 M glacial acetic acid	0.4 M	1.15 mL
3 M sodium acetate	0.4 M	6.67 mL
Ultrapure water	n/a	32.18 mL
Total	n/a	50 mL

*Safety note: Add water first and then slowly add other reagents.*

**9. TBST**

**Table:** 

Reagent	Final concentration	Quantity or volume
10× TBS, pH 7.4	1×	100 mL
100% Tween-20	0.1%	1 mL
Ultrapure water	n/a	900 mL
Total	n/a	1 L

**10. 5% (w/v) non-fat dry milk**

**Table:** 

Reagent	Final concentration	Quantity or volume
Non-fat dry milk	5% (w/v)	10 g
TBST	n/a	200 mL
Total	n/a	200 mL

**11. 20 mM Tris-HCl, pH 7.4**

**Table:** 

Reagent	Final concentration	Quantity or volume
1 M Tris-HCl, pH 7.4	20 mM	10 mL
Ultrapure water	n/a	490 mL
Total	n/a	500 mL

**12. 2% (w/v) sodium azide**

**Table:** 

Reagent	Final concentration	Quantity or volume
Sodium azide	2% (w/v)	4 g
Ultrapure water	n/a	To 200 mL
Total	n/a	200 mL

*Safety note:* ***Only*** *use plastic or non-metal spatula. Wear a mask to avoid inhaling the reagent.*

**13. Primary antibody solution**

**Table:** 

Reagent	Final concentration	Quantity or volume
Mouse anti-FLAG M2 antibody	1:1,000	10 μL
2% (w/v) sodium azide	0.2% (w/v)	100 μL
TBST	n/a	9.89 mL
Total	n/a	10 mL

**14. Secondary antibody solution**

**Table:** 

Reagent	Final concentration	Quantity or volume
HRP-conjugated goat anti-mouse IgG (H+L)	1:30,000	3 μL
TBST	n/a	29.997 mL
Total	n/a	30 mL

**Laboratory supplies**

1. 500 mL vacuum filter with 0.22 μm cellulose nitrate membrane (Corning, catalog number: 430758)

2. 0.2 μm nitrocellulose membranes (Bio-Rad, catalog number: 1620112)

3. BrightStar Plus positively charged nylon membrane (Invitrogen, catalog number: AM10104)

4. 1.7 mL microcentrifuge tubes (Olympus Plastics, catalog number: 24-282)

5. 8-strip PCR tubes (Olympus Plastics, catalog number: 27-125UA)

6. Tissue wipes (VWR, catalog number: 82003-820)

7. Paper towels

8. Plastic wrap

9. 50 mL centrifuge tubes (VWR, catalog number: 89039-656)

10. 15 mL centrifuge tubes (VWR, catalog number: 89039-664)

11. Microcentrifuge tube racks

12. Four-way tube rack

13. Blot development folders (Azure Biosystems, catalog number: AC2126)

14. 5, 10, 25, and 50 mL serological pipettes (Gen Clone, catalog numbers: 12-102, 12-104, 12-106, 12-107)

15. Glass Pasteur pipette

16. Forceps

17. p10, p200, and p1000 pipette tips (VWR, catalog numbers: 76323-388, 76323-390, 76323-456)

## Equipment

1. -80 °C freezer

2. -20 °C freezer

3. 4 °C refrigerator

4. Magnetic rack (DynaMag-2) (Invitrogen, catalog number: 12321D)

5. Slot blot apparatus, Hoefer PR648 (discontinued; often available on eBay) or Bio-Dot SF microfiltration apparatus (Bio-Rad, catalog number: 1703938)

6. Three-way stopcock (Thermo Fisher, catalog number: 64700004)

7. Large plastic container (e.g., Tupperware)

8. p2.5, p10, p20, p200, p1000 pipettes

9. Western blot boxes (with lid)

10. Plastic blot container

11. Eppendorf ThermoMixer F1.5 (Eppendorf, catalog number: 5384000020) or equivalent

12. Eppendorf Centrifuge 5430 (Eppendorf, catalog number: 022620584) or equivalent

13. Benchmark Orbi-Blotter orbital shaker (Benchmark, catalog number: BT30) or equivalent

14. Benchmark BenchRocker 2D (Benchmark, catalog number: BR2000) or equivalent

15. Stratalinker UV Crosslinker 1800 (Stratagene)

16. GelDoc Go Imaging System (Bio-Rad, catalog number: 12009077) or equivalent

17. T100 thermal cycler (Bio-Rad, catalog number: 1861096) or equivalent

18. Azure Sapphire Biomolecular Imager (Azure Biosystems, catalog number: IS4000)

19. Aspirator

20. Millipore ultrapure water system

21. NanoDrop One Microvolume UV-Vis spectrophotometer (Thermo Fisher, catalog number: ND-ONE-W)

22. Benchmark Vortex BenchMixer (Benchmark, catalog number: BV1000) or equivalent

23. Mini centrifuge MLX-106-GS (Genesee Scientific, catalog number: 3377846, 3377848) or equivalent

24. Benchmark Roto-Mini Plus Rotator, variable speed (Benchmark, catalog number: R2024) or equivalent

25. Blotting roller (Thermo Fisher, catalog number: LC2100)

26. 1 L plastic beaker

27. p200 multichannel pipette

## Software and datasets

1. ImageJ (https://imagej.net/ij/, free download from the NIH)

## Procedure

**A. Prepare buffers**

1. Make the following buffers (see Recipes): oligo-dT lysis buffer, oligo-dT wash I buffer, oligo-dT wash II buffer, and oligo-dT elution buffer (TE buffer). Filter each buffer through a 0.22 μm cellulose nitrate (CN) sterilizing vacuum filter.

*Note: Use sterile stir bars or mix using sterile serological pipettes to reduce potential RNase contamination.*

**Critical:** Buffers should be stored at room temperature. We routinely found that buffers perform best when made fresh, approximately every 3 months (see Troubleshooting).

2. Add DTT fresh to a working aliquot of oligo-dT lysis buffer and oligo-dT wash I buffer for each experiment. Aliquot the necessary amount (e.g., oligo-dT lysis buffer: 700 μL per sample; oligo-dT wash I buffer: 1 mL per sample) into a 15 or 50 mL tube, then add 5 mM DTT (final).

**B. Oligo-dT mRNA pulldown**

*Note: All given volumes are for mRNA isolation from a single well of a 6-well plate of cultured cells (approximately 1 × 10^6^ cells). Increase oligo-dT magnetic beads and elution volume 2× for a 10-cm plate and 2.5× for a 15-cm plate. Wash volumes can remain the same as given below but may have to be re-optimized.*

1. Twenty-four hours prior to collection, transfect Neuro2A cells by your method of choice (e.g., Lipofectamine, FuGENE 4K) with plasmids encoding WT and mutant TOP3B-3xFLAG.

2. Lyse cells with oligo-dT lysis buffer (**with freshly added DTT)** for oligo-dT isolation.

*Note: All steps should be performed at room temperature unless otherwise specified.*

a. Twenty-four hours after transfection, when cells are approximately 80% confluent, aspirate off media and gently wash cells in each well with 1 mL of ice-cold PBS. Aspirate PBS.

b. Pipette 500 μL of oligo-dT lysis buffer (**with DTT**) directly into each well. Incubate the plate on an orbital shaker at maximum speed for 10 min at room temperature.

c. Transfer cell lysates to a sterile and pre-labeled 1.7 mL microcentrifuge tube.

*Note: Cell lysates will likely be “goopy” due to the release of genomic DNA. Pipette up and down multiple times in wells before transferring to the microcentrifuge tube to ensure the best transfer of the sample.*

**Pause point:** After transferring to microcentrifuge tubes, samples can be stored at -80 °C until step B2d.

d. Syringe samples 1× with a 28 G needle to reduce sample viscosity (i.e., shear genomic DNA).

**Critical:** Failure to syringe samples may reduce the ability to pull the oligo-dT beads on the magnetic rack and can severely reduce the RNA yield (see Troubleshooting).

*Note: The level of sample viscosity can depend on cell confluency and cell type. For higher confluency cells or different cell types, it may be helpful to syringe 2×. However, we caution against syringing more than 2–3× as this may begin to shear the mRNA and reduce yield.*

3. Prepare oligo-dT magnetic beads and cell lysate for isolation.

a. Label four sterile 1.7 mL microcentrifuge tubes per sample. Place one tube per sample into the removable sample rack of the DynaMag-2 magnet or equivalent. Set aside the three remaining sets of tubes.

b. Bring oligo-dT beads to room temperature, then aliquot 100 μL of oligo-dT magnetic beads into each microcentrifuge tube.

**Critical:** If using a p200 pipette tip to aliquot oligo-dT beads, cut a small portion of the tip off with a **clean** razor blade to reduce potential shearing of beads.

c. Add 200 μL of oligo-dT lysis buffer (**with DTT**) to the beads. Mix by gentle inversion for 2 min. Spin down briefly (~1 s) in a mini centrifuge.

d. Place the samples back into the removable sample rack and place onto a magnetic base for 2 min.

4. Incubate the cell lysate with oligo-dT magnetic beads.

a. Use an aspirator or pipette to remove the supernatant from the oligo-dT magnetic beads while still on the magnetic base.

**Critical:** Do not remove the supernatant until immediately before adding cell lysate onto beads. Allowing beads to dry out can reduce RNA yield.

b. Remove the sample rack from the magnetic base and add the entire volume of prepared cell lysate onto the magnetic beads. Resuspend beads by gently pipetting 5×.

c. Incubate cell lysate with magnetic beads with end-over-end rotation (15 rpm) for 30 min at room temperature.

*Note: Cell lysate incubation can be reduced to 10 min, if necessary; however, we routinely found that 30 min incubation gave slightly higher RNA yield.*

5. Wash oligo-dT magnetic beads.

a. Remove the samples from the rotator and spin down briefly (~1 s) in a mini centrifuge. Place the sample rack on a magnetic base for 2 min and then aspirate and discard the supernatant.

b. Remove the sample rack from the magnetic base. Add 500 μL of oligo-dT wash I buffer (**with freshly added DTT**) to the sample. Resuspend the beads by gently pipetting 4–5×, ensuring that there are no bead clumps remaining. Mix by gentle inversion for 1 min. Spin down briefly (~1 s) in a mini centrifuge. Place the samples on the magnetic base for 2 min, then aspirate and discard supernatant.

c. Repeat step B5b once more for a total of two washes with oligo-dT wash I buffer (**with DTT**).

d. Remove the sample rack from the magnetic base. Add 500 μL of oligo-dT wash II buffer to the sample. Resuspend beads by gently pipetting 2–3×, ensuring that there are no bead clumps remaining. Mix by gentle inversion for 1 min. Spin down briefly (~1 s) in a mini centrifuge. Place the samples on the magnetic base for 2 min, then aspirate and discard the supernatant.

e. Repeat step B5d once more for a total of two washes with oligo-dT wash II buffer.

f. Remove the sample rack from the magnetic base. Add 500 μL of oligo-dT low salt buffer to the sample. Resuspend the beads by gently pipetting 2–3×, ensuring that there are no bead clumps remaining. Mix by gentle inversion for 1 min. Spin down briefly (~1 s) in a mini centrifuge. Place the samples on a magnetic base for 2 min, then aspirate and discard the supernatant.

*Note: We routinely observe that the magnetic beads pull more slowly to the magnet following the addition of oligo-dT low salt buffer. This should not impact RNA yield.*

6. Elute oligo-dT isolated RNA from beads and remove the magnetic beads from the sample.

a. Remove the sample rack from the magnetic base. Add 200 μL of oligo-dT elution buffer to the sample. Resuspend the beads by gently pipetting 2×, then transfer the resuspended beads to one set of the pre-labeled sterile 1.7 mL microcentrifuge tubes that were set aside at step B3a.

b. Briefly (~2–3 s) vortex samples on a low speed.

c. Heat samples at 50 °C with agitation (300 rpm in Thermomixer) for 2 min.

d. Briefly spin samples down (~1 s) in a mini centrifuge. Place the sample rack onto the magnetic base for 2 min. **DO NOT discard supernatant as it contains the isolated mRNA**.

e. While samples are still on the magnetic base, transfer 195 μL of sample into the second set of pre-labeled sterile 1.7 mL microcentrifuge tubes that were set aside at the beginning (step B3a). Be careful to avoid magnetic beads during transfer. After transferring your sample, tubes containing the remaining magnetic beads can be discarded.

f. Spin samples at 18,000 rcf for 5 min at room temperature in a microcentrifuge (e.g., Eppendorf Centrifuge 5430) to remove any residual magnetic beads.

**Critical:** Skipping this step can cause residual magnetic beads to remain in your sample and lead to small “splotches” of signal across your slot blot. Residual magnetic beads in the sample can also interfere with mRNA quantitation via UV spectroscopy (see Troubleshooting).

g. Transfer 185 μL of sample to the final pre-labeled set of 1.7 mL microcentrifuge tubes that were set aside at the beginning (step B3a). Immediately place samples on ice. **This is your final sample.**

**Pause point:** Samples can be used immediately or stored long term at -80 °C.

**C. Slot blotting, day 1**

1. Prepare and equilibrate nitrocellulose and nylon membranes for slot blotting.

a. Cut one nitrocellulose membrane and two nylon membranes to size with a clean pair of scissors or razor blade. Cut the bottom left corner of all membranes for orientation.

b. Incubate the nitrocellulose and nylon membranes in 20 mM Tris-HCl, pH 7.4, for 1 h at room temperature. P1000 pipette tips can be used to ensure that the entire membrane is submerged beneath the buffer.

2. Measure concentration and prepare RNA dilutions for slot blotting.

a. Determine concentration of isolated mRNA from previous steps using UV spectroscopy with a Nanodrop One Spectrophotometer (see General notes).

b. In a sterile PCR strip, dilute all mRNA samples to 2 μg of RNA in 1× sterile PBS in a total volume of 200 μL. Mix by gentle inversion, briefly spin in a mini centrifuge, and heat-denature at 70 °C for 15 min in a thermal cycler.

**Figure 1.**
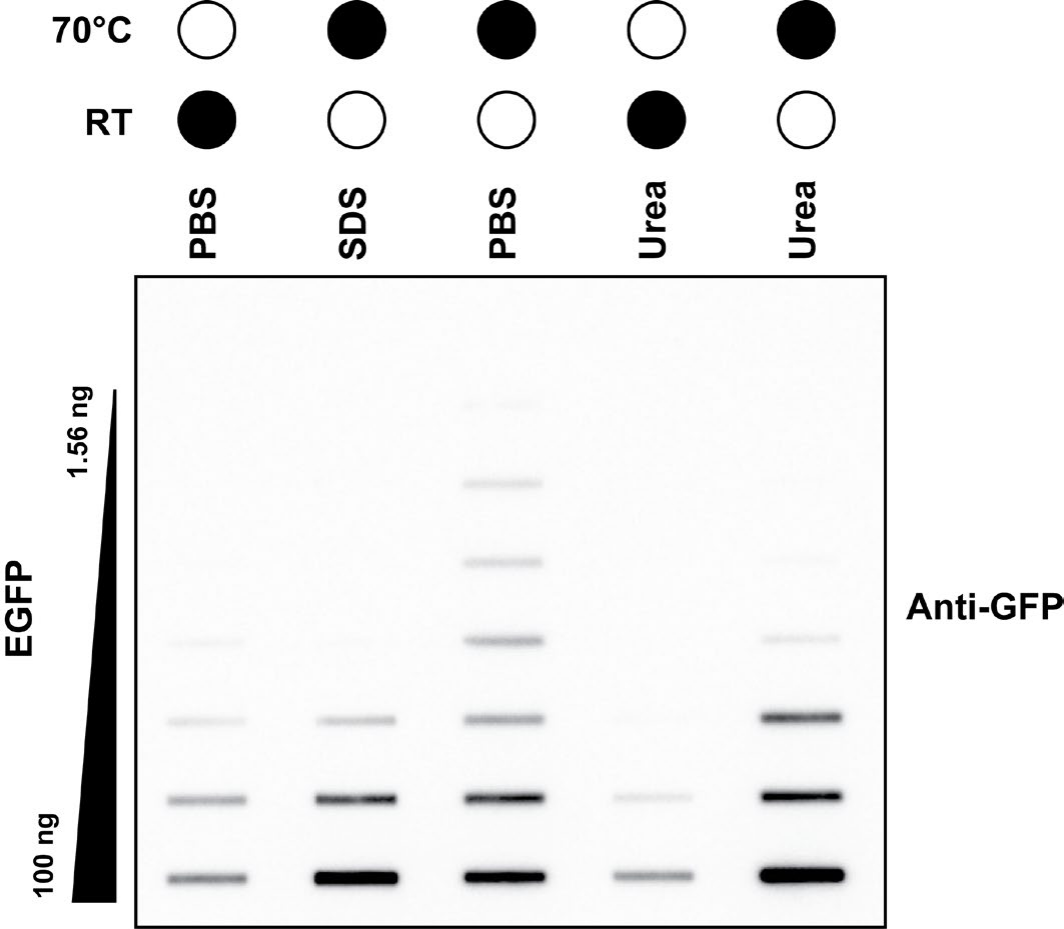
Heat denaturing allows the largest dynamic range of detection. Anti-GFP slot blot of a two-fold dilution titration of recombinant EGFP protein. Samples were diluted in either 1× PBS, 4% (w/v) SDS, or 8 M urea and then incubated at room temperature (RT) for 30 min or at 70 °C for 15 min. Black filled circles represent the temperature of sample incubation.

*Note: We routinely found that heating at 70 °C for 15 min resulted in the largest dynamic range of recombinant EGFP detection compared to other denaturing methods tested (Figure 1). This may differ between other proteins of interest and other antibodies, especially if the chosen antibody recognizes native/folded protein better. This may need to be empirically determined. For TOP3B, the C-terminus that harbors 3xFLAG is predicted to be largely disordered [14]. However, it is known that SDS interferes with protein binding to nitrocellulose membranes.*

**Figure 2.**
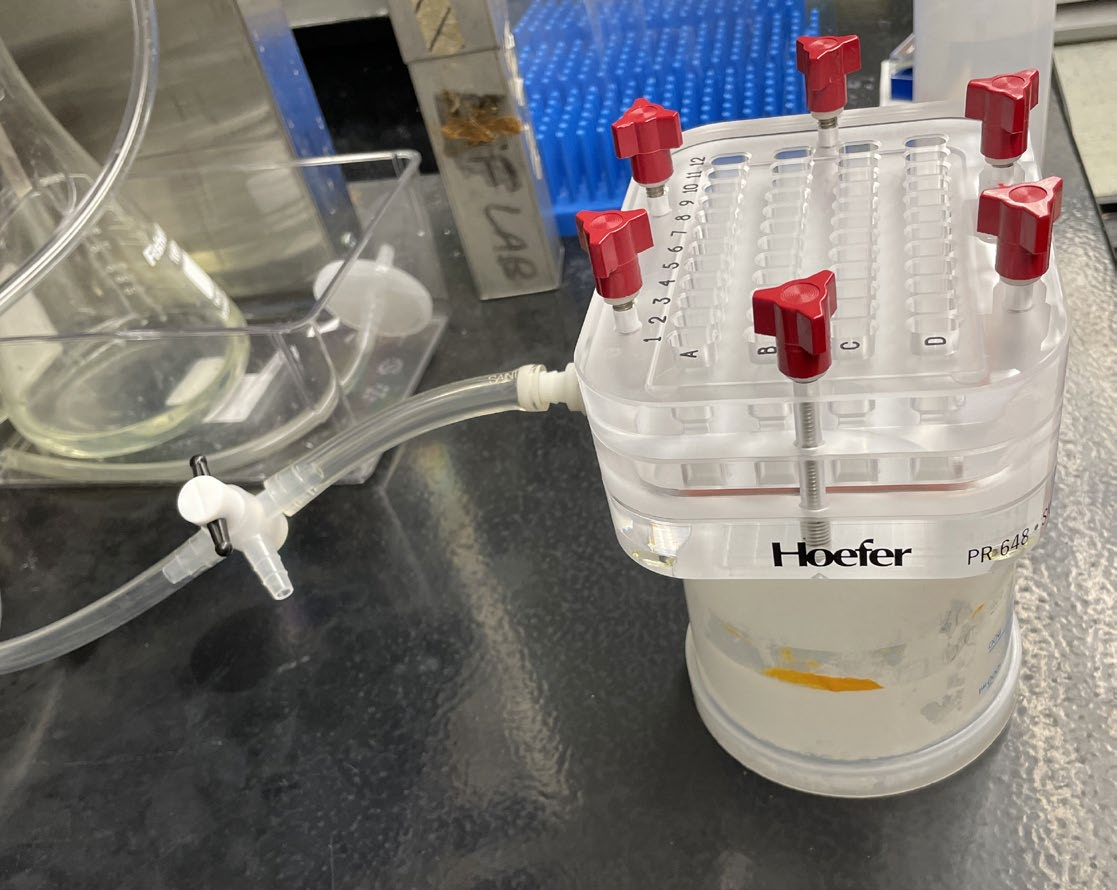
Example of slot blot apparatus setup. A slot blot apparatus placed on a raised surface (e.g., an upside-down 1 L plastic beaker) and attached to a vacuum line with a three-way stopcock air valve.

**Figure 3.**
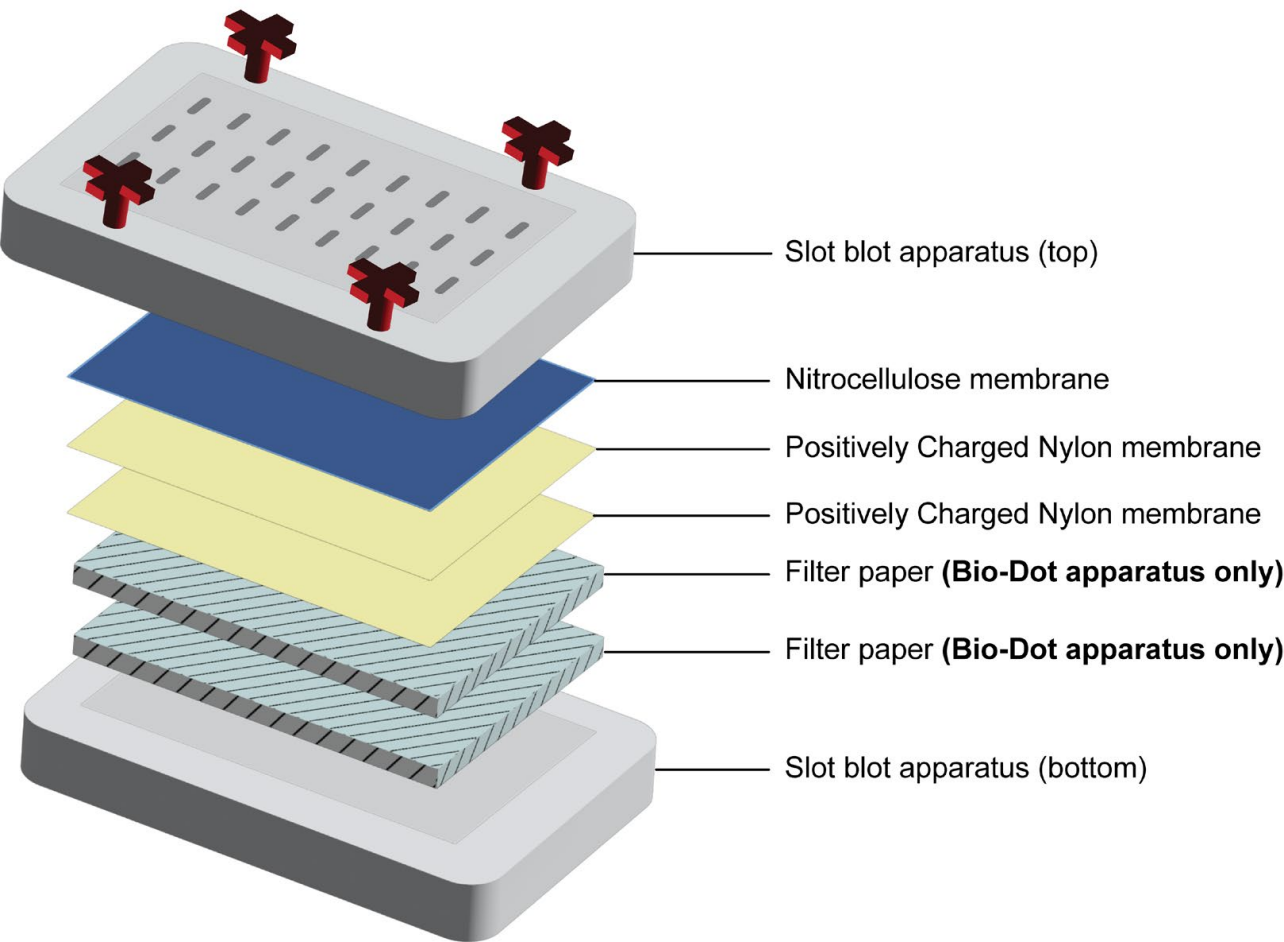
Diagram showing the membrane order for the slot blot setup. Onto the bottom piece of the slot blot apparatus, two pieces of filter paper should be placed **only when using the Bio-Dot apparatus**. For all slot blots, two pieces of positively charged nylon membrane, followed by 1 nitrocellulose membrane, should be placed before securing the top slot blot piece in place.

3. Assemble slot blot apparatus.

a. Place slot blot apparatus on a raised platform and attach the slot blot apparatus, the three-way stopcock air valve, and the hose to a standard house/building vacuum source (Figure 2). Turn on the vacuum but leave the air valve closed (see General notes).

b. Remove the top piece of the slot blot by loosening the screws and place it onto a clean paper towel or Kimwipe. Place two nylon membranes followed by one nitrocellulose membrane onto the slot blot apparatus using forceps. The nitrocellulose membrane should be on top. Between the addition of each membrane, use a blot roller or a clean serological pipette to remove bubbles and center membranes on the apparatus. If using the Bio-Rad slot blot apparatus, two pieces of filter paper (equilibrated in 20 mM Tris-HCl) should be added below the nylon and nitrocellulose membranes (Figure 3).

*Note: Upon loading the sample and applying vacuum, TOP3B•mRNA covalent intermediates will bind the nitrocellulose membrane on top, and the free mRNA will flow through and bind to the nylon membrane underneath. Two pieces of nylon are used to reduce the diffusion of the sample that can interfere with methylene blue staining.*

c. Use a Kimwipe to remove any buffer from the edges of the slot blot apparatus. Leaving buffer around the edges can interfere with forming the required seal.

d. Place the top slot blot piece onto the apparatus. Tighten screws in an X pattern, then tighten two side screws if applicable (e.g., tighten in the order of top left, bottom right, top right, bottom left, two side screws last). Only hand-tighten; do not use a wrench.

*Note: The Bio-Rad slot blot apparatus does not have the two side screws, but it should still be tightened in the X pattern.*

4. Pre-wash slot blot membranes with 20 mM Tris-HCl, pH 7.4.

a. Using a multichannel pipette, add 195 μL of 20 mM Tris-HCl, pH 7.4, to the slot blot apparatus slots. Vacuum through by turning the air valve to full vacuum. Repeat for a total of two washes.

*Notes:*

*1. All slots on the slot blot must be filled with buffer for the apparatus to work properly.*

*2. If the buffer appears to be leaking immediately after addition, tighten the screws slightly with the vacuum turned on.*

*3. Turn the air valve off as soon as the buffer is through to avoid drying out the membrane prior to loading samples. This can cause a dark ring around the slotted sample after developing your blot.*

5. Load the mRNA sample onto the slot blot and wash.

a. With the air valve closed, and using a multichannel pipette, add 195 μL of mRNA sample (from step C2b) onto the slot blot apparatus. To the remaining open slots not filled with sample, add 195 μL of 20 mM Tris-HCl. Vacuum samples through.

*Note: If possible, we recommend loading your first sample in lane 2 (rather than lane 1) for easier marking and cutting of the membrane later.*

b. Wash membranes once with 195 μL of 20 mM Tris-HCl. Vacuum through.

6. Disassemble the slot blot apparatus.

a. Leaving the air valve open (vacuum on), loosen the screws of the slot blot apparatus in the reverse order that they were tightened. Remove the top part from the slot blot apparatus and place it onto a clean paper towel or Kimwipe.

b. Immediately after removing the top piece, mark the slots surrounding your samples with a pencil to aid with trimming the membrane later. For example, if samples are in lanes 2–6, you would mark lane 1 and lane 7 with a pencil.

7. Remove the nitrocellulose membrane, block in 5% (w/v) non-fat dry milk (see Recipes), and incubate in primary antibody.

a. Leaving the vacuum on, use forceps to carefully remove the nitrocellulose membrane and place it into a small container or blot box containing 5% (w/v) non-fat dry milk.

b. Block the membrane in non-fat dry milk for 30 min with shaking on an orbital shaker at room temperature.

c. Wash 3× in TBST (see Recipes) to remove excess non-fat dry milk.

d. Place a nitrocellulose membrane on a plastic wrap sheet, then fold the plastic wrap over the membrane to create a folder. Using a **clean** razor blade, cut the membrane around the previously made pencil markings. Cut one corner of each membrane for orientation.

**Critical:** Use a brand-new razor blade or one that has been carefully cleaned with 70% ethanol. Failure to do so will cause background during immunodetection (see Troubleshooting).

**Critical:** We highly recommend cutting off the entire perimeter edge of the nitrocellulose membrane prior to antibody incubation, especially if using the Bio-Rad slot blot apparatus. We routinely noticed that failure to remove these edges led to background during immunodetection around the entire perimeter of the blot.

e. Remove the cut slot blot from the plastic wrap and place it into a blot incubation box with a lid containing your diluted primary antibody. In the example below and with the plasmids described above, we use mouse anti-FLAG M2 antibody at a 1:1,000 dilution (see Recipes).

f. Incubate blots in primary antibody overnight on a rocker at 4 °C.

8. Remove the top nylon membrane from the slot blot apparatus and stain with methylene blue.

a. Leaving the vacuum on, use forceps to carefully remove the top nylon membrane and place it on a clean paper towel. The remaining bottom nylon membrane (and filter papers if used) can be discarded.

b. With the top nylon membrane on the paper towel, crosslink the mRNA onto the membrane. We routinely use the autocrosslink setting on a Stratalinker UV Crosslinker 1800, which is 120,000 µJ/cm^2^.

c. Incubate the nylon membrane in methylene blue staining solution (see Recipes) with shaking on an orbital shaker for 20 min at room temperature. Be sure to completely cover the membrane with the stain.

d. Remove and save the methylene blue staining solution (it can be reused). Wash the nylon membrane with distilled water on an orbital shaker until sufficient background staining has been removed. The time depends on how fresh the methylene blue staining solution is. However, two washes of approximately 1–2 min each are typically sufficient.

e. Image the methylene blue–stained nylon membrane on a Bio-Rad gel doc or an equivalent instrument. **This serves as a loading control**.

9. Clean the slot blot apparatus.

a. Once all membranes are removed, the vacuum can be turned off. Dismantle the apparatus. Clean the middle and top parts of the slot blot apparatus with a small amount of standard dish soap and distilled water. The bottom layer can just be rinsed with distilled water. The Bio-Rad slot blot can be left to air dry, but the Hoefer slot blot should be fully dried with a Kimwipe. Leaving the acrylic wet can cause it to swell and lose its ability to seal.

**D. Slot blotting, day 2**

**Figure 4.**
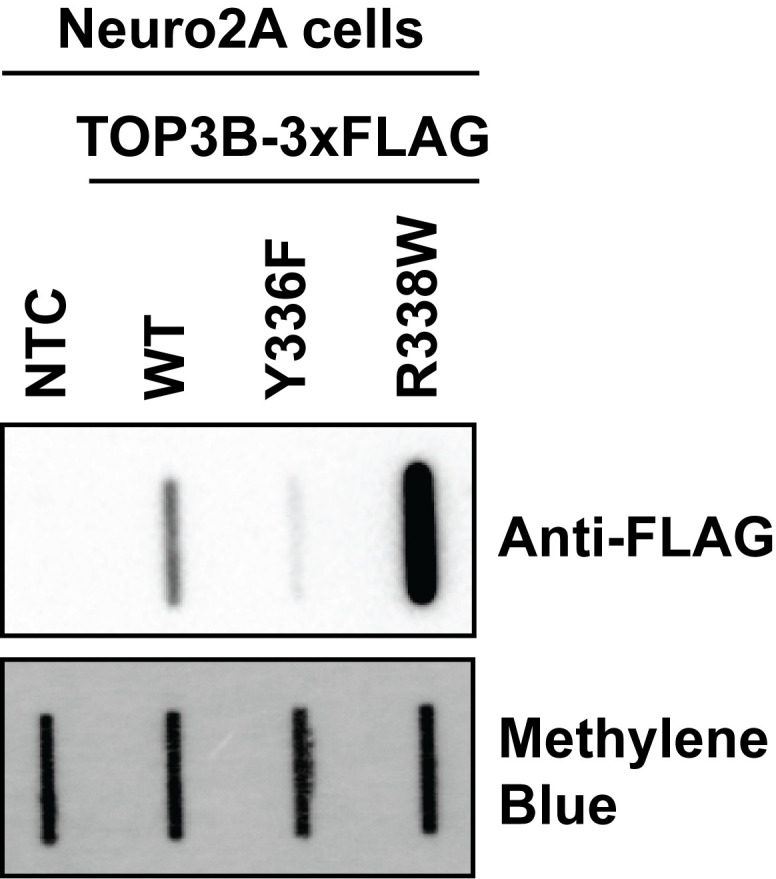
Oligo-dT pulldown selectively isolates TOP3B•mRNA covalent intermediates. Anti-FLAG slot blot of wildtype (WT) and mutant TOP3B•mRNA covalent intermediates isolated using denaturing oligo-dT magnetic bead pulldown from Neuro2A cells (nitrocellulose membrane). Compared to WT, covalent intermediates produced by the catalytically inactive mutant (Y336F) are reduced, but those produced by the synthetic self-trapping mutant (R338W) are robustly detected. Free mRNA was stained with methylene blue (positively charged nylon membrane) and served as a loading control.

1. Wash the nitrocellulose membrane 3 × 10 min with TBST: Remove the nitrocellulose membrane from the primary antibody and place it into the blot box containing ~30 mL of TBST. Incubate with shaking on an orbital shaker for 10 min at room temperature. Repeat this wash twice for a total of three washes.

2. Incubate the nitrocellulose membrane in secondary antibody solution for 1 h at room temperature: Remove the last TBST wash, add secondary antibody, and incubate on an orbital shaker for 1 h at room temperature. Here, we used goat anti-mouse secondary antibody conjugated to HRP at a 1:30,000 dilution (see Recipes).

3. Wash the nitrocellulose membrane 3 × 10 min with TBST as in step D1.

4. Using clean forceps, pick up the nitrocellulose membrane from one corner, allow it to drip dry for 5–10 s, and then place it on a flat piece of plastic film.

5. Develop chemiluminescence by incubating membranes with 2 mL of prepared West Pico SuperSignal Plus for 5 min at room temperature. Briefly drip dry to remove excess reagent and transfer to a clean plastic sleeve or plastic wrap for imaging. Capture chemiluminescence using Azure Sapphire or equivalent (Figure 4).

## Data analysis

After chemiluminescence imaging, TOP3B•mRNA covalent intermediate levels can be quantitated by standard densitometry using ImageJ [15,16]. A mock-transfected/no-template control or an empty plasmid control should be included to ensure that the immunodetection is specific. Signals between conditions and/or mutants can then be compared using standard statistical tests (e.g., Student’s t-test with Welch correction for two samples and a one-way ANOVA with Dunnett’s multiple comparisons for three or more samples). We also recommend including the Y336F catalytic inactive mutant and the “self-trapping” R338W mutant as additional controls, as these should produce the lowest and highest signals, respectively. At least three biological replicates should be used for all samples and conditions.

## Validation of protocol

This protocol has been validated in Warrick et al. [14]. An autism spectrum disorder mutation in Topoisomerase 3β causes accumulation of covalent mRNA intermediates by disrupting metal binding within the zinc finger domain. *Nucleic Acids Res*. 53(20). DOI: 10.1093/nar/gkaf1138. See Figures 1, 2, 3, and 6, and Supplementary Figures S1, S2, S3, and S5.

## General notes and troubleshooting

**General notes**

1. We recommend using a three-way stopcock to better control the vacuum power and prevent diffusion of the sample on the nitrocellulose membrane when the vacuum is off. This produces sharper bands on the final blot, which can help with ImageJ analysis.

2. We recommend using an empty vacuum flask to reduce splashing into the vacuum line when opening and closing the three-way stopcock that is connected to the vacuum.

3. From one well of a 6-well plate, oligo-dT pulldown should yield a concentration ranging from 30 to 50 ng/μL. However, RNA concentration will depend on cell type and confluency.

4. Perform dilutions of mRNA input to ensure that detection is in the linear dynamic range. TOP3B-R338W produces very robust covalent intermediates, and its detection could be saturated. This may affect the interpretation of results.

5. This protocol can be performed using cells expressing truncations or point mutations in the protein of interest to identify important regions for mRNA binding.

6. This application can also be used in conjunction with other methods, such as immunoprecipitation and RNA-seq, to identify sites of protein binding. This method can also be used to evaluate the association of other species with your protein of interest, as was done by Warrick et al. [14] to determine the association of ubiquitin with TOP3B•mRNA covalent intermediates.

7. While the slot blot is a faster method that allows for the addition of more sample in a highly concentrated area to increase signal compared to a western blot, a limitation of this approach is the inability to determine the molecular weight and ensure that the full-length protein is being detected.

8. It should be noted that recommend buffers do not explicitly eliminate potential RNA scaffolds. However, high-throughput sequencing of RNA isolated by crosslinking immunoprecipitation (HITS-CLIP) and enhanced crosslinking immunoprecipitation (CLIP) provided evidence that TOP3B binds directly to mRNA [4,9].

**Troubleshooting**

**Problem 1:** Low signal on slot blot.

Possible causes: Buffers were made more than three months ago and have “gone bad.” Poor transfection can also lead to lower signal.

Solutions: Make fresh buffers and repeat the protocol. Be sure to always add DTT fresh to a working aliquot of the oligo-dT buffers. Confirm the desired transfection efficiency.

**Problem 2:** Background around the edge or in the middle of the blot during immunodetection.

Possible causes: Residual oligo-dT magnetic beads were not adequately removed prior to slot blotting, or the razor blade used to cut the membrane was not clean.

Solutions: Ensure you are removing residual magnetic beads from eluted mRNA by centrifugation at 18,000 rcf for 5 min (see step B6f). If background persists, increase spin time and ensure you are not touching the pipette tip to the residual bead pellet. Use a brand-new razor blade to cut the nitrocellulose membrane.

**Problem 3:** Beads are not pulling to the magnetic rack.

Possible causes: The starting samples may be too viscous. We have noted that certain cell drug treatments can cause this. Variation in buffers can also cause this.

Solutions: Syringe samples more before addition to beads. Use suggested buffers within three months of making them.

**Problem 4:** Samples will not vacuum through the slot blot apparatus.

Possible cause: Bubbles in the slot blot wells can interfere with the vacuum and limit sample flow.

Solution: Turn off the vacuum, use a pipette tip to gently pop all bubbles, and turn the vacuum back on. Check if the vacuum power is 19–25 inHg.

**Problem 5:** Negative control has high signal.

Possible cause: Wash conditions are not stringent enough.

Solutions: Ensure complete mixing during every bead wash step. If necessary, increase time spent gently pipetting to mix the beads with each wash buffer and/or add an additional incubation step with wash buffers and end-over-end rotation. Check if the buffers were made correctly. If the problem persists, increasing LiCl and/or LiDS in the oligo-dT lysis buffer and oligo-dT wash I buffer may help. However, this may result in changes to the yield.
